# Graphene Quantum Dots: Green Synthesis, Characterization, and Antioxidant and Antimicrobial Potential

**DOI:** 10.1155/2024/2626006

**Published:** 2024-01-23

**Authors:** Pooja Kadyan, Ponnusamy Thillai Arasu, Sudhir Kumar Kataria

**Affiliations:** ^1^Department of Zoology, Maharshi Dayanand University, Rohtak, Haryana, India; ^2^Department of Chemistry, College of Natural and Computational Sciences, Wollega University, P.O. Box 395, Nekemte, Ethiopia

## Abstract

Antibiotic resistance is becoming more common, emphasising the demand for novel antibacterial treatments. The current investigation describes the green synthesis of graphene quantum dots (GQDs) using *M. indica* leaves and characterization via Fourier-transform infrared spectra (FTIR), X-ray diffraction (XRD), scanning electron microscope (SEM), transmission electron microscope (TEM), and ultraviolet-visible (UV-Vis) spectrophotometer. The results showed that GQDs are spherical in shape. *In vitro* antioxidant and antimicrobial studies indicate that the biological efficacy of synthesized GQDs was higher than the ethanolic leaf extract. GQDs exhibited the highest scavenging efficacy with lowest IC_50_ (half-maximal inhibitory concentration) value. However, antimicrobial study showed more inhibitory activity of GQDs against all screened microorganisms, i.e., *Staphylococcus aureus*, *Escherichia coli*, *Bacillus subtilis*, and *Pseudomonas aeruginosa*, and fungi, i.e., *Aspergillus niger* and *Aspergillus flavus*. Graphene quantum dots facilitate reactive oxygen species (ROS) which ultimately lead to antioxidant and antibacterial activity. This approach would provide an efficient alternate method for tackling microorganisms.

## 1. Introduction

According to the Centers for Disease Control and Prevention (CDC), around two million people are infected with antibiotic-resistant bacteria each year, and approximately 23,000 people die as a direct result of these infections [1]. The prevention and cure of these illnesses has gotten a lot of attention, and it is a huge challenge to discover medicines, antibiotics, and/or antibacterial chemicals that can suppress bacterial development. *Pseudomonas aeruginosa* and *Staphylococcus aureus* infections have been reported in surgical regions, where they are associated to skin damage and burned areas [1]. The bacteria that are Gram-negative have a lipid-rich exterior membrane, a plasma membrane, and a thin peptidoglycan layer, whereas Gram-positive bacteria have thicker layer, less susceptible to damage of cell walls [2]. This variation in composition of cell walls is essential for antibiotic research as bacterial resistance may be caused partly by cell wall and its composition [3]. To combat infections caused by bacteria, numerous antibacterial medicines such as streptomycin, tetracycline, and sulphonamides have been created. These antibiotics, however, have failed to kill numerous kinds of bacteria, and strains that are multidrug-resistant have appeared as a result of pathogen evolution in overcoming the biocidal activity of the agent molecules. As a result, antibacterial agents that can overcome the restrictions of typical antibacterial agents and, ideally, fight against both types of bacteria must be developed. Microbial infections are seen as a serious public health issue, with an increasing focus on those which do not react to treatment due to antibiotic-resistant bacteria. During the post-antibiotic period, carbon-based nanomaterials with unique properties could provide innovative solutions. Emerging materials include carbon-based nanomaterials, i.e., graphene oxide, graphene nanosheets, reduced graphene oxide, graphene quantum dots, etc. [4]. Numerous innovative research areas have been established as a consequence of rapid advancements in nanotechnology and nanoscience. Graphite, diamonds, graphene oxide (GO), fullerenes, carbon nanotubes (CNTs), carbon dots, carbon quantum dots, and graphene quantum dots (GQDs) are some of the conventional allotropes of carbon-based nanomaterials that have gathered prominence due to their different applications in the energy, environmental, and healthcare sectors owing to their electrical, optoelectronic, and catalytic properties [5–7]. Carbon-based materials have sparked research interest in various fields because of their outstanding properties, like large surface area, tunable structure, high conductivity, less toxicity, and long life [8]. GQDs are considered one of the recent entrants that garnered significant research interest due to their low cytotoxicity, responsiveness to chemicals, stable fluorescence properties, high hydrophobicity, biocompatibility, photocatalytic activity, photostability, low cost, and photoluminescence properties [9, 10]. GQDs are also used in LEDs, biosensors, drug delivery, solar cells, tissue engineering, bioimaging, photodynamic therapy, batteries, and photocatalysis [10–12]. GQDs can be synthesized by “bottom-up” and “top-down” approaches via different synthetic procedures. GQDs can be synthesized using biological approaches rather than using harsh, toxic, or expensive chemical compounds. Green synthesis is more favourable than other approaches to synthesize GQDs due to the easier availability of biomass [13]. GQDs are praised by many researchers due to their intriguing properties, including low cytotoxicity, excellent water solubility, high electrical conductivity, good biocompatibility, chemical stability, photoluminescence, low photobleaching, environmental friendliness, and optoelectronic properties. GQDs have the potential to be used in flash memory devices, solar cells, electronic displays, packaging, LEDs, antibacterial activity, drug delivery, tissue engineering, supercapacitors, batteries, optoelectrical detectors, bioimaging, photodynamic therapy photocatalysis, anticancer agents, various biosensors, lithium-ion batteries, and energy conversion applications [12]. In India, traditional experts are gradually shifting to different kinds of medicinal plants to treat various diseases. Mango has great medicinal value and is a worldwide distributed plant [14]. Mango plant is mostly used in traditional medicine in many therapeutic activities and it grows well mainly in tropical and semitropical countries. Mango leaves are easily available and are nontoxic natural materials. Mango extracts have antibacterial, antifungal, antiviral, anthelmintic, antiallergic, gastroprotective, antiplasmodial, and anti-inflammatory properties [15]. To the best of our knowledge, no research paper has been published yet on the antibacterial and antioxidant properties of GQDs derived from mango leaves. In this study, we report the microwave-assisted green synthesis of GQDs using mango leaves. Free radicals or reactive oxygen species (ROS) are responsible for inflammation because they act upon various biological molecules, and by taking out electrons to enter into a stable state, they exert damage. The use of plant leaves for the fabrication of graphene quantum dots has numerous benefits, including being widely available, affordable, safe, demanding no use of toxic chemicals, and having a wide range of metabolites that are involved in the production of GQDs.

## 2. Materials and Methods

### 2.1. Plant Material Identification

The leaves of *Mangifera indica* were collected from the Herbal Garden of Maharshi Dayanand University, Rohtak, which were further identified and authenticated by CSIR-NIScPR, New Delhi (Specimen voucher Ref. No. NIScPR/RHMD/2022/4021-22-2). The leaves were then washed with deionized water to remove dust particles and shade-dried for 12 days. The dried leaves were ground to obtain a fine powder.

### 2.2. Chemicals and Reagents

2,2-Diphenyl-1-picrylhydrazyl, butylated hydroxyl toluene, sodium phosphate, ammonium molybdate, gallic acid, Muller–Hinton agar, Muller–Hinton broth, streptomycin, rifamycin, Sabouraud dextrose agar, Amphotericin B Disc, 7-hydroxy-3H-phenoxazin-3-one-10-oxide, ascorbic acid, ethanol, methanol, and sulfuric acid of analytical grade were purchased from HiMedia.

### 2.3. Microorganism

The test organisms included Gram-negative bacteria: *P. aeruginosa* (MTCC 424) and *E. coli* (MTCC 443), Gram-positive bacteria: *S. aureus* (MTCC 96) and *B. subtilis* (MTCC 121), and two fungi: *Aspergillus niger* (MTCC 281) and *Aspergillus flavus* (MTCC 277). They were procured from the Department of Microbiology and Biotechnology of Maharshi Dayanand University, Rohtak, India.

### 2.4. Preparation of Plant Extract

The shade-dried leaf powder of *M. indica* was extracted with ethanol by the maceration method. In brief, 20 g of powder was dissolved into 200 ml absolute ethanol and kept at room temperature for 48 h. The resultant was then filtered using Whatman filter paper no. 1, and for further use, the dried extracts were kept at 4°C [16].

### 2.5. Green Synthesis of GQDs

**Figure 1:**
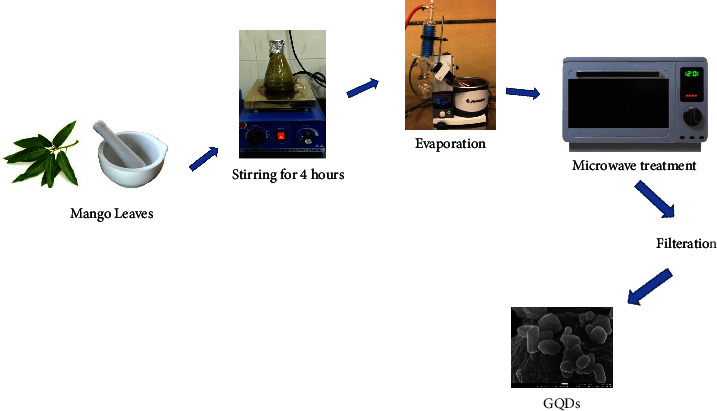
Green synthesis of GQDs using mango leaves.

Green synthesis of GQDs was done following the protocol of Kumawat et al. with some modifications [17]. 20 g of *M. indica* leaves powder was mixed in 200 ml of absolute ethanol and kept for 4 h with constant stirring. Then, the extract was centrifuged at speed of 5000 rpm for 15 min to obtain clear solvent. Afterward, the solvent was concentrated by a rotary evaporator to obtain the slurry. Then, 2 ml of Milli-*Q* water was mixed with the obtained thick slurry, and the mixture was heated at 900 W in a microwave oven for 5–8 min. Afterward, the residue was dispersed into analytical-grade ethanol and then filtered by using a syringe filter to obtain pure GQDs (Figure 1). For further use, the dried powder was stored at 4°C. Synthesized GQDs were characterized using a UV-Visible spectrophotometer, TEM, SEM, FTIR, XRD, and Zeta potential.

### 2.6. Characterization of GQDs

To study size, morphology, and elemental analysis, different characterization techniques were used such as SEM, TEM, FTIR, EDX, Zeta potential, UV-Visible spectrophotometer, and fluorescence spectrophotometer. UV-Visible spectroscopy and fluorescence spectroscopy using Shimadzu (UV 3600 plus) and Horiba (KL3C-21) were done to detect emission and excitation spectra at Aryabhata Central Instrumental Laboratory (ACIL), M.D. University, Rohtak. The diffraction pattern of GQDs was analysed using Rigaku (SmartLab 3 KW). To get the morphology and elemental analysis, FESEM-EDX analysis was done by using JEOL (7610F plus) electron microscope at Guru Jambheshwar University, Hisar. HRTEM study was conducted to get the size of GQDs using TECNAI (200 kv) at Sophisticated Analytical Instrument Facility for Electron Microscopy, AIIMS, New Delhi. FTIR analysis was used to identify the functional group in synthesized GQDs using Bruker Alpha II infrared spectrophotometer at M.D. University, Rohtak. To detect particle size and stability of synthesized GQDs, Zeta sizer analysis was done using Malvern (Nano ZS) at ACIL, M.D. University, Rohtak.

### 2.7. *In Vitro* Antioxidant Assay

#### 2.7.1. DPPH Free Radical Scavenging Assay

The antioxidant potential of the mango leaf extract and synthesized GQDs was assessed by 2,2-diphenyl-1-picrylhydrazyl scavenging assay [18]. The DPPH method is an easy, simple, and precise method. We mixed 2 mL of test samples (20, 40, 60, 80, and 100 *µ*g/mL) into 2 mL of 0.05 mg/mL DPPH ethanol solution. The antioxidant's DPPH can attach to H atom of GQDs and form a stable H-DPPH complex with change in a colour while the concentration of DPPH was measured at 517 nm. The percentage inhibition was evaluated by the given formula

(1) $$\begin{array}{c} \%\text{DPPH}\;\text{radical}\;\text{scavenging}=\frac{\left(\text{Aa}-\text{Ab}\right)}{\text{Aa}}\times 100, \end{array}$$

where Aa = absorbance of the control and Ab = absorbance of the sample.

#### 2.7.2. Phosphomolybdenum Assay

The antioxidant activity of green synthesized GQDs and plant extract was examined using phosphomolybdate in acidic pH according to Sharadanand Phatak and Hendre with slight modifications [19]. 0.2 ml of sample solution (GQDs and plant extract) treated with phosphomolybdate reagent (0.588 ml sulfuric acid, 0.049 g ammonium molybdate, and 0.036 g sodium phosphate) was incubated for 90 minutes at 95°C. The absorbance was measured at 695 nm. Phosphomolybdate reagent with methanol acted as a control. Gallic acid was used as a standard.

#### 2.7.3. Agar Well Diffusion Assay

The antimicrobial sensitivity activity of prepared GQDs and leaf extract was evaluated by the agar well diffusion method [20]. Bacteria were cultured in Muller–Hinton broth, and the turbidity was matched to 0.5 McFarland standards. The inoculum was prepared by inoculating the nutrient broth and incubated at 37 C in shaking incubator for 24 h. The culture was diluted with 0.9% saline solution to obtain 1.5 × 10^8^ CFU/ml. Each culture was spread with the help of sterilized swab on MHA plates separately. By following inoculation, wells were punched using a cork borer and wells were loaded with 100 *µ*L of samples (6.25, 12.5, 50, and 100 mg/ml). Streptomycin and rifamycin were used as a positive control. After the incubation of 24 h at 37°C, the zone of inhibition for different samples was measured.

#### 2.7.4. Disc Diffusion Assay

The antifungal activity of prepared GQDs and leaf extract was evaluated by the disc diffusion method [21]. Sabouraud dextrose agar media plates were inoculated with cultures of microbial inocula. With the help of a sterile forceps, sterile discs (6 mm in diameter) were placed. GQDs and plant leaf extracts of various concentrations (6.25, 12.5, 50, and 100 mg/ml) were applied on the discs. Amphotericin B disc was used as positive control. After the incubation of 24 h at 37°C, the zone of inhibition for different samples was measured.

#### 2.7.5. MIC Assay

In order to define MICs (minimum inhibitory concentrations) which can stop growth of bacteria, we choose a situation in which there is a colour change of resazurin. The test samples were mixed with MHB and were introduced to column 1 and gradually diluted till column 11. Each well received 10 *μ*L of standard microbial solution, leading to 10^5^ CFU/ml. Column 12 contained solely the bacterial suspension and acted as a control. After 24 hours, 10 *μ*L of 0.5 mg/ml resazurin in DI had been added to every well (from rows A to D), and the resulting cultures were left for incubation of 3 hours at 37°C. A shift in colour from blue to a pink colour demonstrates bacterial activation of resazurin, and that MIC was established as a minimal concentration of test samples that inhibited resazurin colour change.

### 2.8. Statistical Analysis

The experiments were repeated three times. The obtained results were statistical analysed by using GraphPad Prism 8.2 software. The experimental data were subjected to one-way analysis of variance with Tukey test at a 95% confidence interval. Each value represents the mean ± SD wherein *P* value represents level of significant changes. The XRD and UV-Visible spectra were plotted in “Origin Pro 2023.”

## 3. Results and Discussion

**Figure 2:**
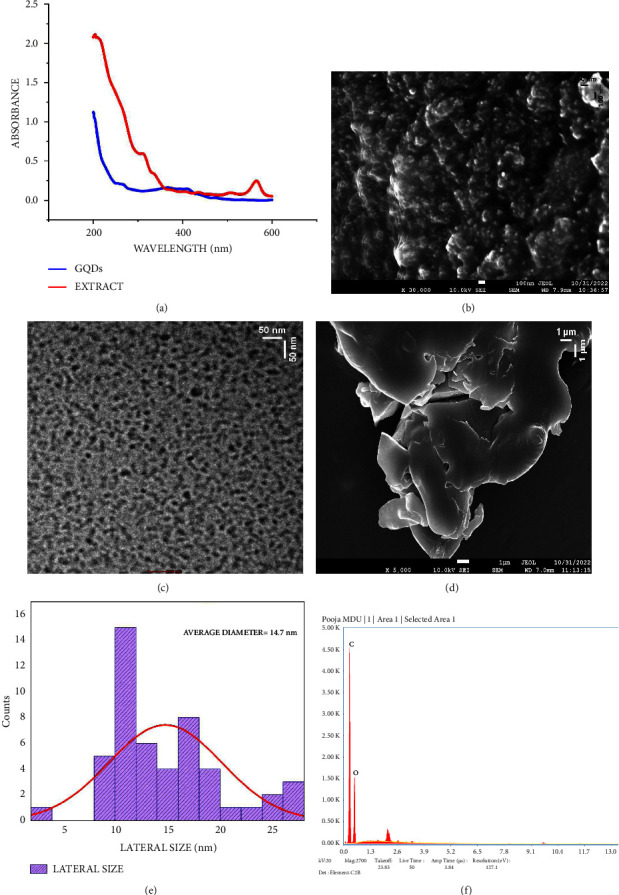
(a) Absorption spectra, (b) SEM image at low magnification, (c) TEM image, (d) FESEM image at high magnification, (e) size distribution image, and (f) elemental analysis (EDX spectra) of biosynthesized GQDs.

A continuous absorption spectrum was obtained for synthesized GQDs.  Figure 2(a) exhibits similar absorbance peaks at 260 nm (attributed to the presence of *π*-*π*^*∗*^ transition of C-C bond and because of sp^2^ domain) and 362 nm (*n*-*π*^*∗*^ transition of C=O bond) as reported in the previous study [22, 23]. The structural morphology of GQDs was observed by TEM. The sample solution was treated in an ultrasonic bath before deposition to decrease aggregation. A drop of evenly dispersed GQDs alcoholic solution was deposited on copper grid. The samples were dried and analysed at an acceleration voltage of 200 kV and resolution of 0.24 nm. Figures 2(b)–2(d) illustrate the formation of GQDs which appear spherical in shape. Particle size distribution histogram determined from the TEM images (Figure 2(e)) showed average size of 14.7 nm. The presence of elements in the analysed structures was also confirmed by the application of EDX measurements. The amount of each element detected by EDX is shown in Figure 2(f). Carbon and oxygen were detected on the surface of GQDs, thereby indicating the presence of several O-containing functional groups.

### 3.1. FTIR Analysis

**Figure 3:**
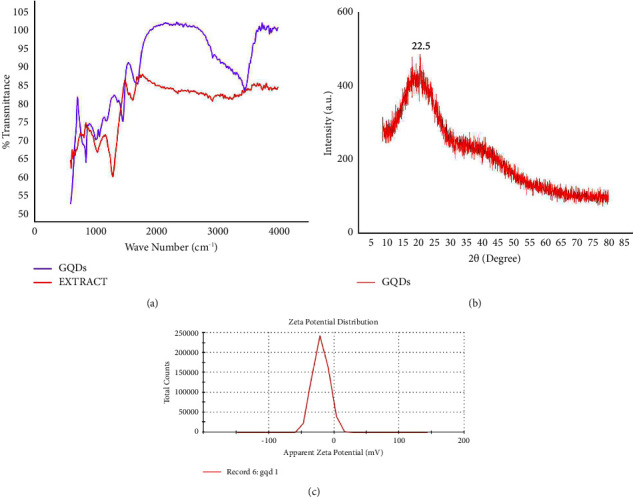
(a) FTIR spectra, (b) XRD pattern, and (c) Zeta potential of synthesized GQDs.

FTIR is an effective method to distinguish different types of functional groups in plant extracts and nanomaterials by analysing chemical bonds. To identify functional groups that are present in green synthesized GQDs, FTIR spectroscopy was carried out. FTIR analysis showed shifts in the absorbance peak of GQDs with a range varying from 500 to 4000 cm^−1^ as shown in Figure 3(a). IR bands observed at 3455.5 cm^−1^ could correspond to N-H stretching vibrations. The observed band at 2933.2, 1685.3, 1443.4, 1188.4, and 835.9 cm^−1^ for GQDs could emerge due to -CH stretching, C=C O-H bending, C-O stretching, and C-N stretching vibration, respectively. The spectrum reflects high density of hydroxyl groups and oxygenated carbon groups present on the edges of the GQDs. FTIR peaks were observed by Shi et al. at 1378 cm^−1^, 1091 cm^−1^, and 3445 cm^−1^ [22]. The IR band at 2927.33, 2358.5, 1616.2, and 1031 cm^−1^ for the extract could correspond to CH stretching, -C=C stretching, aromatic C=C (unsaturated ketone), and NH_2_ group.

### 3.2. XRD Analysis

The study was done at 2*θ* of a range from 10 to 80°. XRD data of synthesized GQDs showed the presence of GQDs through the peak at 22.5 as depicted in Figure 3(b). Moreover, there are no other peaks found in the XRD spectrum of GQDs that could be related to any impurity, which suggests that the synthesized material is highly pure. XRD analysis was used to check the crystallographic information of synthesized GQDs.

(2) $$\begin{array}{c} D=\frac{0.94\lambda }{\beta \mathrm{Cos}\theta }, \end{array}$$

where *λ* is the wavelength (1.5406), *θ* is the Bragg angle of diffraction, and *β* represents line broadening at half of maximum intensity.

The XRD pattern of GQDs appears as a weak broad peak centered around 2*θ* = 22.5°. This peak was assigned to the (002) plane of graphitic carbon, revealing a graphitic nature of the GQDs with highly disordered carbon atoms. This supports the hypothesis that the synthesized GQDs have a graphitic structure with a small amount of amorphous carbon that have been fabricated and agrees well with the previously reported works [22–24].

### 3.3. Zeta Potential

The Zeta potential of prepared GQDs showed a negative value of −20.2 mV as shown in Figure 3(c). Furthermore, the observed Zeta potential validated the GQD surface's negatively charged nature. This also suggests the presence of deprotonated carboxylic acid groups on the surface of GQDs. Furthermore, particles are said to be stable if they have a strong Zeta potential (positive or negative). Based on the FTIR spectra and Zeta potential measurements in this study, the GQDs were shown to be negatively charged in Zeta potential measurements due to the presence of many hydroxyl and carboxyl groups.

### 3.4. DPPH Radical Scavenging Assay

**Figure 4:**
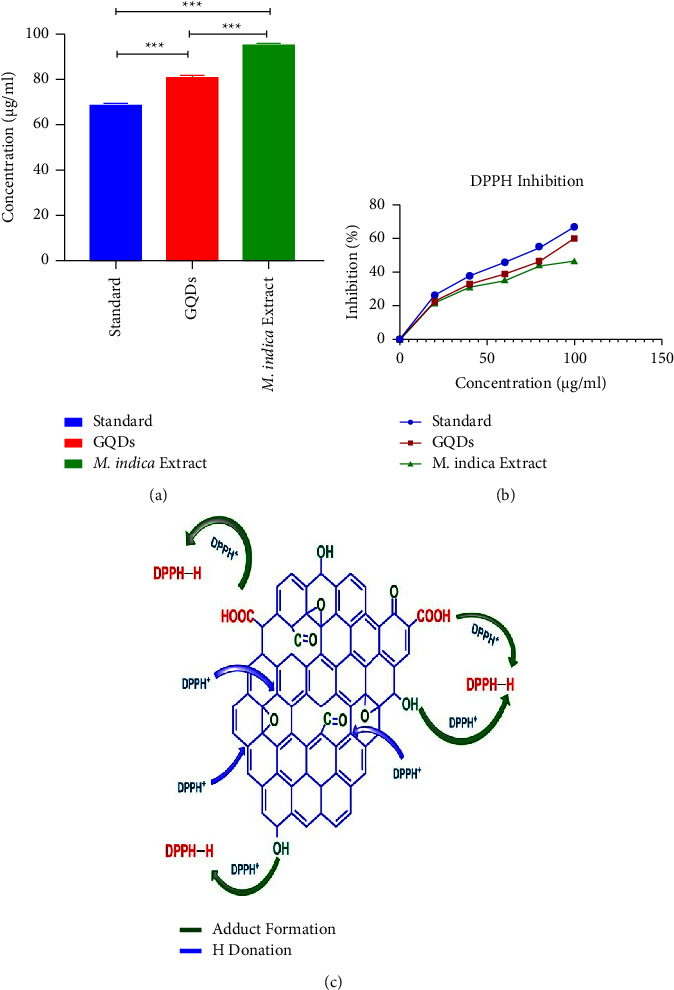
Antioxidant potential of GQDs and extract: (a) comparison of IC_50_ value of GQDs and extract with ascorbic acid, (b) comparison of % inhibition with ascorbic acid, and (c) hypothetical antioxidant mechanism of GQDs.

The antioxidant activity of the *M. indica* leaf extract and synthesized GQDs was assessed by DPPH scavenging assay. Various concentrations of the sample (20, 40, 60, 80, and 100 *μ*g/ml) were prepared in analytical grade methanol. The primary indication is the change in colour which is the measure of free radical scavenging. The results showed that the active scavenging activity increases in a dose-dependent manner, and a summary of ANOVA is shown in Table 1. Synthesized GQDs exhibit high antioxidant activity as compared to the leaf extract as shown in Figures 4(a) and 4(b). A proposed mechanism for radical scavenging is linked to the transfer of hydrogen from GQDs surfaces to DPPH. Unpaired electrons on the GQD surface (Figure 4(c)) can be distributed via chemical bond rearrangement. The existence of hydroxyls (-OH), carboxyls (-COOH), and amino groups (-NH_2_ and -NH) enables the transfer of hydrogen atom and the reduction of DPPH [25]. The IC_50_ of an antioxidant-containing material is the concentration required to scavenge 50% of the initial DPPH radicals. The IC_50_ value is inversely proportional to the free radical scavenging activity/antioxidant property of the sample. The lesser the IC_50_ value is, the better the compound is at scavenging DPPH, implying greater antioxidant activity. GQDs have less IC_50_ value than the extract, hence better antioxidant activity than the crude extract.

### 3.5. Total Antioxidant Capacity of GQDs

The phosphomolybdate assay is based on the principle of reduction of molybdenum at low pH by forming a green colour complex. The greater the intensity of green colour, the higher the value of antioxidants present in the sample [26]. The leaf extract of mango was established to possess a significant total antioxidant capacity than GQDs (312.1 > 248.78 mgGAE/g) due to the presence of polyphenols in the leaf extract.

### 3.6. Antimicrobial Activity

**Figure 5:**
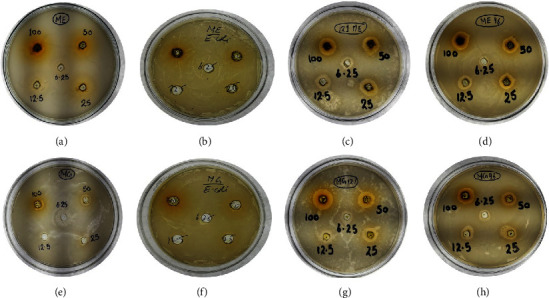
Antibacterial sensitivity testing: zone of inhibition of mango extract against (a) *P. aeruginosa* (MTCC 424), (b) *E. coli* (MTCC 443), (c) *B. subtilis* (MTCC 121), and (d) *S. aureus* (MTCC 96) and zone of inhibition of mango GQDs against (e) *P. aeruginosa* (MTCC 424), (f) *E. coli* (MTCC 443), (g) *B. subtilis* (MTCC 121), and (h) *S. aureus* (MTCC 96) at different concentrations.

**Figure 6:**
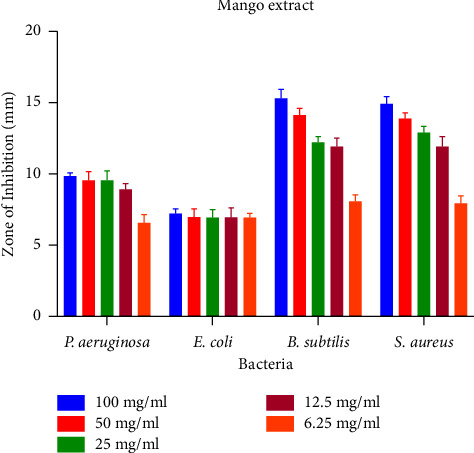
Zone of inhibition of mango extract at different concentrations against various bacteria.

**Figure 7:**
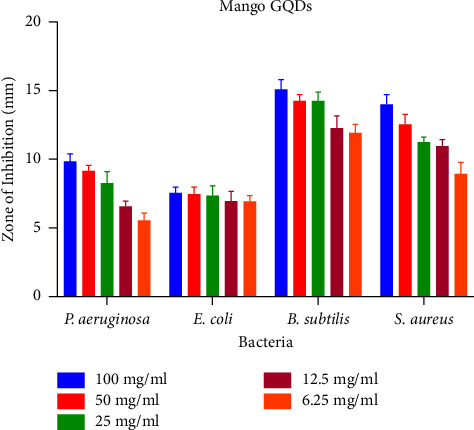
Zone of inhibition of mango graphene quantum dots (GQDs) at different concentrations against various bacteria.

**Figure 8:**
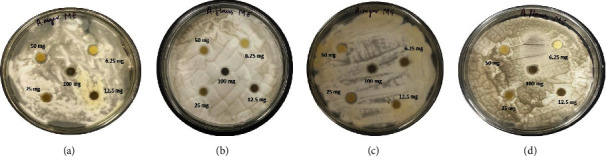
Antifungal sensitivity testing: zone of inhibition of mango extract against (a) *A. niger* (MTCC 281) and (b) *A. flavus* (MTCC 277) and zone of inhibition of mango GQDs against (c) *A. niger* (MTCC 281) and (d) *A. flavus* (MTCC 277) at different concentrations.

Different concentrations of synthesized GQDs and leaf extracts were prepared (100, 50, 25, 12.5, and 6.25 mg/ml) for the determination of antibacterial activity. A zone of inhibition of antibacterial capacity that ranges from 7.01 ± 0.57 mm to 15.4 ± 0.22 is depicted in Figure 5. No zone of inhibition was produced by the leaf extract and synthesized GQDs against *E. coli* (MTCC 443). The strongest antimicrobial activity was found to be 15.4 mm in GQDs against *B. subtilis.* GQDs and mango leaf extract also showed antibacterial activity against *S. aureus* (MTCC 96). The results of antibacterial sensitivity test are shown in Table 2 and Figures 6 and 7. Furthermore, zone of inhibition that appeared in *Aspergillus flavus* (MTCC 277) was 1.9; however, GQDs showed a maximal zone of inhibition of 2.4 ± 0.34 at 100 mg/ml against *Aspergillus niger* (MTCC 281) (Figure 8). Based on MIC results (Table 2), synthesized GQDs have better antimicrobial activity than the crude leaf extract. Lowest MIC was observed for *S. aureus* (MTCC 96), i.e., 0.391 mg/ml, and highest MIC was observed against *E. coli* (MTCC 443) which is more than 100 mg/ml.

### 3.7. Possible Antibacterial Mechanistic Approach

**Figure 9:**
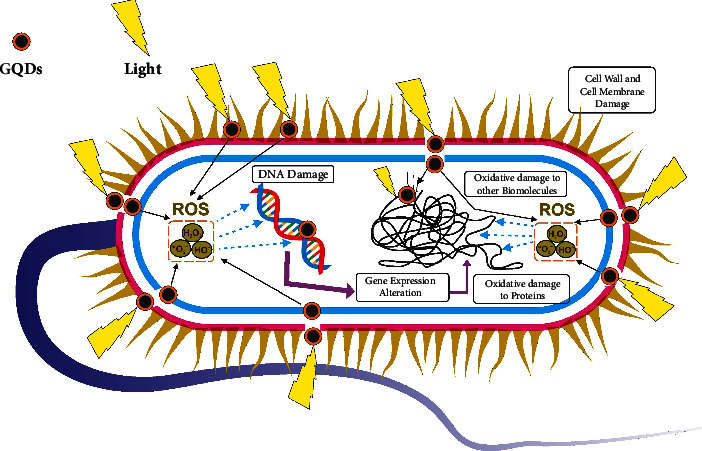
Hypothetical mechanism of GQDs for antibacterial activity [29].

The mechanism of GQDs and other graphene-based nanomaterials as an antibacterial agent is not completely understood; however, various possible mechanisms have been documented which include the following. Graphene-based substances with a smaller dimension and greater number of functional groups have a huge chance of interacting with bacterial cells which leads to cell deposition. GQDs may cause membrane stress by breaking cell membranes which results in cell death [27, 28]. Some research suggests that graphene-based materials can cause superoxide anion-independent oxidative damage in bacterial cells (Figure 9). The mechanism primarily involves the material sticking to the bacterial cell wall, followed by the release of the reactive oxygen species (ROS), which included production of free electrons which reacted with different molecules in the cell to generate free radicals. These radicals destroyed DNA and hindered its replication process, after which proteins were damaged and oxidised, influencing microbial metabolism. The never-ending cycle continues, resulting in the bacterial population being eradicated. GQDs reduced bacterial growth *via* a complex method. GQDs' antibacterial effectiveness was greatly dependent on their surface charge as well as the production of reactive oxygen species.

## 4. Conclusion

Green synthesis of GQDs using plant leaf extracts is a promising method to obtain eco-friendly nanomaterials having various biological applications. Microwave-assisted green synthesis method was successfully done for the synthesis of GQDs. The microwave-assisted method has various advantages like rapid heating, low cost, shorter reaction time, and being environmentally friendly. Hence, the green synthetic approaches might be an alternative to other methods which indicates the possibility of the plant in the development of various products for biological applications. The green synthesis of GQDs by using different plant parts is an area to be explored more. Synthesized GQDs were found to possess significant antioxidant and antimicrobial activities. Because of their ease of manufacturing and functionalization, substantial water solubility, and apparent biocompatibility, GQDs are attractive antibacterial nanomaterials. The study not only revealed the material's potential for multiple applications but also offered new insight into how to use low-cost garbage in a way that is more ecologically sound. We anticipate that using the knowledge gained in this study, the physicochemical features of graphene-based materials, such as number of functional groups, size, and conductivity, can be better adjusted to either reduce hazards or increase application possibilities.

## Figures and Tables

**Table 1:** Statistical analysis of antioxidant activities of leaf extract and GQDs.

ANOVA summary	Result
*F*	4488
*P* value	<0.0001
Significant differences among mean (*P* < 0.05)	Yes
*R* square value	0.9993

**Table 2:** Zone of inhibition (ZOI) and minimum inhibitory concentration (MIC) of GQDs against different microbes.

S. No	Microbes	ZOI of GQDs (100 mg/ml)	ZOI of mango extract (100 mg/ml)	MIC (mg/ml)
1	*S. aureus*	15.0 ± 0.2	14.2 ± 0.3	0.391
2	*P. aeruginosa*	9.9 ± 0.2	9.3 ± 0.3	12.5
3	*E. coli*	7.2 ± 0.4	7.1 ± 0.2	>100
4	*B. subtilis*	15.4 ± 0.2	15.1 ± 0.4	0.781
5	*Aspergillus flavus*	1.9 ± 0.4	1.4 ± 0.2	12.5
6	*Aspergillus niger*	2.4 ± 0.2	2.1 ± 0.3	25

## Data Availability

The data supporting this study will be available on request.

## Additional Points

*Highlights*. (i) A facile and green synthesis of GQDs was demonstrated using the microwave-assisted method. (ii) GQDs are characterized via SEM, TEM, Zeta potential, UV-Visible spectrophotometer, and fluorescence spectrophotometer. (iii) The green GQDs showed higher antioxidant and antimicrobial activity than the crude leaf extract. (iv) The antimicrobial activity and its possible mechanism were also discussed.
